# Current practices and barriers impairing physicians’ and nurses’ adherence to analgo-sedation recommendations in the intensive care unit - a national survey

**DOI:** 10.1186/s13054-014-0655-1

**Published:** 2014-12-05

**Authors:** Barbara Sneyers, Pierre-François Laterre, Marc M Perreault, Dominique Wouters, Anne Spinewine

**Affiliations:** Université catholique de Louvain, Louvain Drug Research Institute, Clinical Pharmacy Research Group, Bâtiment Van Helmont - Avenue Mounier, 72 boite B1.72.02-1200, Bruxelles, Belgium; Université catholique de Louvain, Cliniques universitaires Saint Luc, Department of Pharmacy, Avenue Hippocrate, 10-1200, Bruxelles, Belgium; Université catholique de Louvain, Cliniques universitaires Saint Luc, Department of Intensive Care and Emergency medicine, Bruxelles, Belgium; Faculty of Pharmacy, Université de Montréal, Montreal, Canada; Université catholique de Louvain, CHU Dinant-Godinne, Department of pharmacy, Yvoir, Belgium

## Abstract

**Introduction:**

Appropriate management of analgo-sedation in the intensive care unit (ICU) is associated with improved patient outcomes. Our objectives were: a) to describe utilization of analgo-sedation regimens and strategies (assessment using scales, protocolized analgo-sedation and daily sedation interruption (DSI)) and b) to describe and compare perceptions challenging utilization of these strategies, amongst physicians and nurses.

**Methods:**

In the 101 adult ICUs in Belgium, we surveyed all physicians and a sample of seven nurses per ICU. A multidisciplinary team designed a survey tool based on a previous qualitative study and a literature review. The latter was available in paper (for nurses essentially) and web based (for physicians). Topics addressed included: practices, perceptions regarding recommended strategies and demographics. Pre-testing involved respondents’ debriefings and test re-test reliability. Four reminders were sent.

**Results:**

Response rate was 60% (898/1,491 participants) representing 94% (95/101) of all hospitals. Protocols were available to 31% of respondents. Validated scales to monitor pain in patients unable to self-report and to monitor sedation were available to 11% and 75% of respondents, respectively. Frequency of use of sedation scales varied (never to hourly). More physicians than nurses agreed with statements reporting benefits of sedation scales, including: increased autonomy for nurses (82% versus 68%, *P* <0.001), enhancement of their role (84% versus 66%, *P* <0.001), aid in monitoring administration of sedatives (83% versus 68%, *P* <0.001), and cost control (54% versus 29%, *P* <0.001). DSI was used in less than 25% of patients for 75% of respondents. More nurses than physicians indicated DSI is contra-indicated in hemodynamic instability (66% versus 53%, *P* <0.001) and complicated weaning from mechanical ventilation (47% versus 29%, *P* <0.001). Conversely, more physicians than nurses indicated contra-indications including: seizures (56% versus 40%, *P* <0.001) and refractory intracranial hypertension (90% versus 83%, *P* <0.001). More nurses than physicians agreed with statements reporting DSI impairs patient comfort (60% versus 37%, *P* <0.001) and increases complications such as self-extubation (82% versus 69%, *P* <0.001).

**Conclusions:**

Current analgo-sedation practices leave room for improvement. Physicians and nurses meet different challenges in using appropriate analgo-sedation strategies. Implementational interventions must be tailored according to profession.

**Electronic supplementary material:**

The online version of this article (doi:10.1186/s13054-014-0655-1) contains supplementary material, which is available to authorized users.

## Introduction

Minimization of sedatives is recognized as the cornerstone of sedation management in ICUs, and strategies targeting light sedation – including assessment using validated scales, protocolization and daily sedation interruption (DSI) – are associated with improved patient outcomes [1-4]. However, which strategy is safest or most efficient is still a subject of debate, so the choice of management depends on the context and potential barriers identified locally [1,5,6]. Similarly, appropriate pain assessment and management is associated with important patient benefits [7,8]. Despite support from various guidelines to assist clinicians in implementing these practices in ICUs, the use of such strategies varies internationally and shows substantial room for improvement [1,9-11]. Furthermore, observational studies have shown that nearly one-half of ICU patients are deeply sedated [12,13].

Research looking specifically at factors influencing practices in the field of analgo-sedation in the ICU is limited [14-21]. Some studies were qualitative in nature, limiting generalizability of results [18-20]. Quality improvement projects have provided valuable information on how to improve practices; unfortunately these studies were typically monocentric or conducted in a limited number of centers, thereby also reducing generalizability [22-26]. Finally, the body of evidence has come exclusively from North America; the European perspective, with its own barriers and enablers, has not been explored.

Implementing analgo-sedation strategies requires daily commitment and strong collaboration of all clinicians involved, especially nurses and physicians. Lack of shared understanding regarding these practices is present within ICU teams [13,14,18,20]. We have conducted a qualitative study suggesting that profession is a key factor in influencing adherence to analgo-sedation recommendations [20]. ‬Previous research has mostly focused on the physician’s or the multidisciplinary team’s perspectives [11,14,15,17,27]. The nursing’s perspectives were underrepresented in multidisciplinary surveys to assess heterogeneity between professions. Identifying barriers for physicians and nurses separately and specifically is essential to design interventions for improvement, tailored to each profession.

Our first objective was to describe reported utilization of: analgesics and sedatives; validated scales for pain assessment; validated scales for sedation assessment; protocolized analgo-sedation; and DSI. A second objective was to describe the presence of common perceptions enabling or challenging utilization of these strategies (more particularly assessment of sedation using validated scales and DSI) and compare these amongst physicians and nurses.

## Materials and methods

We conducted a nationwide survey on sedation and analgesia practices and their determinants in ICUs across Belgium.

### Target population and sampling frame

All nurses and physicians working in an adult ICU composed our target population. One hundred and one hospitals in Belgium were identified as having an adult ICU. Neonatal, pediatric and subacute ICUs were excluded, as analgo-sedation practices and guidelines differ in those settings. We estimated the size of our total target population and calculated the minimal sample required to ensure a maximum of 5% sampling error, at a 95% confidence interval [28]. The minimal sample size was 224 and 345 responses from physicians and nurses, respectively. Assuming a 50% response rate, targeting at least 448 physicians and 690 nurses was required to achieve adequate power for both populations. Our sampling frame therefore included all physicians and seven nurses (chief nurse and six other nurses) per hospital.

Existing databases did not contain complete and reliable information on our target population and sampling frame. To create the sampling frame database, we conducted a preliminary survey, addressed to all human resources’ departments (HRDs) identified from the governmental website for public health. Contact information for the nurses and physicians of our sampling frame was requested in this preliminary survey. HRDs were also provided with a probabilistic sampling rule to select nurses. Additional data collection for the preliminary survey included: region, academic status of the hospital, number of ICUs and ICU beds, type of activity (medical, surgical, medico-surgical), and staffing information. HRD responses were encouraged using one email and one telephone reminder.

### Survey instrument construction

A multidisciplinary team including an ICU physician (PFL), an ICU nurse (Giuseppe Tirone), two ICU pharmacists (BS, MMP) and a sociologist with previous experience in healthcare surveys (Vincent Lorant), was involved to construct the survey tool (the French version of the survey tool is available in Additional file 1). We identified important questionnaire items through a literature review and a previous qualitative study [11,20,29]. To ensure face and content validity, team members were asked to comment on relevance of items and response choices, and whether these should be included in the questionnaire. Those most pertinent were retained after consensus. Issues regarding terminology and interpretation of the questions were also discussed.

The survey instrument was structured into five sections: use of sedation protocols and sedation scales, reported indications and common perceptions regarding their uses and effects; use of DSI, its contraindications and common perceptions regarding its use; the main analgesia and sedation regimens used; the main strategies regarding analgesia assessment; and demographic data for respondents and their practice setting. Questions regarding practices were multiple choice; for those regarding common perceptions, participants were to indicate agreement with statements using six-point Likert scales. The questions referred to participants’ perceptions of their own everyday practice and to the patients they cared for in their ICUs.

### Survey instrument pretesting

We pretested the survey instrument with one physician, two nurses and one pharmacist using respondent debriefings [30]. Several aspects of the instrument and cover letter were explored, including the layout, format, attractivity, ordering of questions, respondent burden, and understanding of each question (appropriateness of vocabulary, background knowledge and of response categories). Also, 12 respondents (four physicians and eight nurses) completed the survey twice within a 2-week interval. Test–retest reliability yielded Cohen's kappa values >0.40, representing moderate to good agreement [31].

### Survey dissemination

As the survey instrument was constructed in French, it was translated to Dutch and further appraised by two Dutch-speaking ICU practitioners (one physician, one nurse). Both survey instruments (French and Dutch) were available in paper and in an electronic version, using Survey Monkey version 9.1 software (SurveyMonkey Inc., Palo Alto, California, USA). The electronic version was tested on various email addresses, resolving informational technologies issues. A prelabeled, stamped envelope was provided to respondents to return the paper survey questionnaire. Four reminders were sent to each group within 2 months of the first invitation (March to May 2011). A cover letter was provided with the survey tools. The letter contextualized and described the objectives of the survey, mentioned support from professional national societies (the national Belgian Society for Intensive Care (SIZ), its nursing section (SIZ-Nursing) and the College of Physicians for Intensive Care), assured participants of the confidentiality of their responses, and mentioned the results of the survey would be provided to those responding. Nurses were provided with the paper survey questionnaire, as most lacked a professional email address and computers were unavailable to most nurses during working hours. A link to the web-based version was emailed to physicians, because their professional email address was provided through the HRD. If physicians had not responded after the fourth reminder, a paper version was sent to them, using their postal address.

### Ethical concerns

A central ethics committee (Université Catholique de Louvain, Cliniques Universitaires St Luc, Brussels, Belgium) approved our research protocol. Participation in the survey was voluntary; participants’ written consent was not requested as it was implied by completion of the questionnaire. The cover letter guaranteed confidentiality of the responses provided. The respondents received a coded questionnaire. The code was used only to provide reminders to nonresponders. Additionally, participants could withdraw from the web-based survey at any time, as a ‘quit the survey’ option was available on all pages of the web-based survey.

### Statistical analysis

To describe practices and demographic characteristics, we used descriptive statistics including mean values and standard deviations for continuous variables, and frequencies and percentages for categorical variables. Nurses’ and physicians’ characteristics were compared using chi-square tests for categorical variables. All analyses were conducted using SPSS (IBM Corp. Released 2011. IBM SPSS Statistics for Windows, Version 20.0. Armonk, NY, USA: IBM Corp.) and P ≤0.05 was deemed significant.

## Results

### Response rate

The overall response rate was 60% (898/1491 participants), representing 94% (95/101) of all hospitals. Response rates were 50% (323/651) and 68% (575/840) for physicians and nurses, respectively. Respondents had diverse professional and hospital characteristics (Tables 1 and 2). We compared characteristics from hospitals with at least one response with those of hospitals from which we received no responses to address respondents’ bias in terms of practice settings. We found no significant differences between both groups for characteristics such as region, academic status, number of hospital beds and type of management (data not shown, available upon request).

**Table 1:** **Respondent demographics**

	**Total, % (** ***n*** **)** ^**a**^	**Physicians, % (** ***n*** **)** ^**b**^	**Nurses, % (** ***n*** **)** ^**c**^
Current function			
Head of ICU	18 (164)	25 (82)	14 (82)
Full time in ICU	57 (508)	39 (126)	66 (382)
Part time in ICU	21 (186)	25 (80)	18 (106)
Resident	0 (5)	2 (5)	–
Other	4 (35)	9 (30)	1 (5)
Experience in ICU			
<2 years	6 (54)	2 (6)	8 (48)
2 to 5 years	14 (126)	11 (37)	15 (89)
6 to 10 years	21 (186)	22 (71)	20 (115)
11 to 20 years	26 (236)	28 (90)	25 (146)
>20 years	27 (243)	21 (69)	30 (174)
Education			
Physicians			
Anesthetist	–	50 (160)	–
Internist	–	26 (84)	–
Cardiologist	–	7 (21)	–
Resident	–	1 (3)	–
Other	–	1 (4)	–
Nurses			
Certified	–	–	8 (46)
Bachelor degree	–	–	18 (103)
Specialized in critical care	–	–	69 (396)
Master in public health	–	–	7 (40)
Other	–	–	2 (9)
Region			
Brussels	14 (122)	14 (44)	14 (78)
Wallonia	40 (364)	38 (122)	42 (242)
Flanders	46 (412)	49 (157)	44 (255)

^a^Number does not total 898 because not all respondents answered each item. ^b^Number does not total 323 because not all respondents answered each item. ^c^Number does not total 575 because not all respondents answered each item.

**Table 2:** **Hospital demographics of respondents**

	**Total**
Hospital type, % (*n*)^a^	
Nonacademic	94 (843)
Academic	6 (55)
ICU type, % (*n*)^a^	
Medical	4 (38)
Surgical	6 (49)
Medico-surgical	90 (799)
Number of hospital beds, % (*n*)^a^	
0 to 250 beds	30 (272)
251 to 750 beds	58 (520)
>750 beds	12 (106)
Number of ICU beds, % (*n*)^a^	
5 to 10 beds	37 (333)
11 to 20 beds	25 (222)
>21 beds	29 (263)
ICU proportion of elective surgery patients, % (*n*)^a^	
<20%	17 (149)
20 to 39%	16 (144)
40 to 59%	25 (222)
60 to 79%	10 (93)
80 to 100%	2 (18)
ICU proportion of mechanically ventilated patients, % (*n*)^a^	
<20%	10 (94)
20 to 39%	27 (240)
40 to 59%	23 (203)
60 to 79%	9 (81)
80 to 100%	1 (8)
Staff (FTE^b^/ICU bed), mean ± standard deviation	
Physicians	0.3 ± 0.3
Nurses^c^	2.1 ± 0.3

^a^Number does not total 898 because not all respondents answered each item. ^b^Staff in full-time equivalents. ^c^Assuming an occupancy rate of 80% of the beds, a 2.1 FTE/bed ratio corresponds approximately to a 0.5:1 nurse/patient ratio.

### Analgo-sedation current practices

The primary analgo-sedation regimen contained an opiate for 88% (713/807) of respondents and sedatives included propofol, midazolam or either of these drugs for 42% (357/856), 16% (139/856), and 42% (360/856) of respondents, respectively. Continuous infusions of sedatives are frequently used: 12% (104/865) of participants used them in >75% of patients, 74% (642/865) used them in 25 to 75% of patients, 13% (116/865) used them in <25% of patients, and only three participants never used them. A majority of respondents (95%, 802/865) reported that midazolam is mainly administered as a continuous infusion. Most participants used morphine and piritramide, as respectively 15% (125/837) and 10% (80/837) of the latter never used these drugs. Only 20% of respondents indicated never using sufentanil and remifentanil (168/835 and 164/840, respectively). Fentanyl and alfentanil were scarcely used: 59% (474/798) and 86% (674/783) of respondents never used such narcotics.

**Figure 1:**
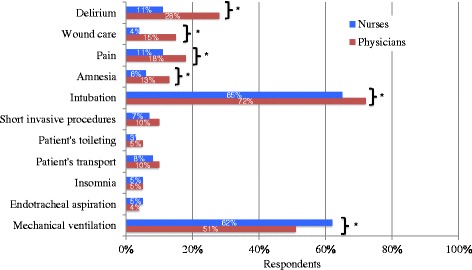
**Perceived indications where sedatives (propofol or benzodiazepines) are frequently used.** To determine indications where sedatives such as propofol or benzodiazepines were used, participants were asked to tick one of the four following choices: ‘never’, ‘rarely’, ‘frequently’ and ‘very frequently’. The answers were compiled in two categories: ‘never or rarely’ and ‘(very) frequently’. The latter category is presented in the figure. *Difference between groups is statistically significant (*P* < 0.05), *P* value calculated using chi-square test.

A majority of respondents reported intubation and mechanical ventilation as indications for sedation (Figure 1). Indications reported for using sedatives varied significantly according to profession. Delirium, wound care, pain, amnesia and intubation were more frequently reported as indications by physicians than nurses, while mechanical ventilation was more frequently reported as an indication for nurses than physicians.‬‬‬‬‬‬‬‬‬‬

Reported availability and utilization of protocolized analgo-sedation, assessment tools for agitation and pain, and DSI are presented in Table 3.

**Table 3:** **Strategies used to assess and treat agitation and pain**

	**Total, % (** ***n*** **)** ^**a**^	**Physicians, % (** ***n*** **)** ^**b**^	**Nurses, % (** ***n*** **)** ^**c**^
Availability of a written analgo-sedation protocol (= Yes)	31 (281)	41 (131)	26 (150)
Frequency of use of analgo-sedation protocols^d^			
Never	6 (16)	3 (4)	8 (12)
<1×/day	27 (76)	21 (27)	33 (49)
1×/day	28 (80)	40 (53)	18 (27)
>1×/day	38 (107)	36 (47)	40 (60)
Availability of a sedation scale (= Yes)	86 (773)	82 (265)	88 (508)
Type of sedation scales used^e^			
RASS	20 (152)	24 (64)	17 (88)
SAS	13 (102)	13 (34)	13 (68)
MAAS	1 (7)	2 (6)	0 (1)
Ramsay sedation scale	64 (491)	69 (184)	60 (307)
GCS	59 (456)	54 (142)	62 (314)
Other sedation scale^f^	13 (103)	9 (24)	16 (79)
Frequency of use of sedation scales on a given patient per day^e^			
Never used, irregularly used, <1×/day	8 (59)	8 (21)	7 (38)
1 to 2×/day	9 (67)	14 (38)	6 (29)
3 to 5×/day	36 (276)	46 (122)	30 (154)
6 to 11×/day	30 (233)	26 (68)	32 (165)
12×/day	13 (98)	3 (7)	18 (91)
> 12×/day	4 (29)	2 (5)	5 (24)
Other	0 (2)	0 (1)	0 (1)
Reported indications for using sedation scales			
To assess the level of sedation	93 (839)	90 (291)	95 (548)
To adjust dosage of sedatives	73 (657)	77 (249)	71 (408)
To assess the level of pain	37 (333)	43 (139)	34 (194)
To adjust dosage of analgesics	33 (299)	38 (123)	31 (176)
Use of daily sedation interruption			
Never	31 (282)	22 (70)	37 (212)
Used in <25% of the patients	44 (393)	38 (122)	47 (271)
Used in 25 to 75% of the patients	17 (149)	25 (80)	12 (69)
Used in >75% of the patients	5 (45)	9 (28)	3 (17)
Opiates are stopped during daily sedation interruption (= Yes)^g^	40 (249)	34 (85)	45 (164)
Assessment of analgesia in patients unable to self-report			
No assessment	7 (59)	4 (12)	8 (47)
Through physiological parameters	88 (787)	80 (257)	92 (530)
Through behavior	85 (765)	81 (263)	87 (502)
During daily sedation interruption	42 (373)	47 (151)	39 (222)
Post-analgesia	55 (492)	46 (150)	59 (342)
BPS	9 (84)	10 (32)	9 (52)
CPOT scale	2 (18)	2 (6)	2 (12)
Other pain scale^h^	19 (172)	9 (30)	25 (142)

ATICE, Adaptation to the Intensive Care Environment, BPS, Behavioral Pain Scale; CPOT, Critical Care Pain Observation Tool; GCS, Glasgow Coma Scale; MAAS, Motor Activity Assessment scale; RASS, Richmond Agitation and Sedation Scale; SAS, Sedation Agitation Scale. ^a^Number does not total 898 because not all respondents answered each item. ^b^Number does not total 323 because not all respondents answered each item. ^c^Number does not total 575 because not all respondents answered each item. ^d^Number totals only those with an analgo-sedation protocol available. ^e^Number includes only those with a sedation scale available within their unit. Some respondents have indicated the use of more than one scale. ^f^Respondents were asked to indicate any other scale used (for example: ATICE, 1% (7); Bloomsbury, 2% (14); Brussels, 2% (18)). ^g^Number totals only those using daily sedation interruption. ^h^Respondents were asked to indicate any other scale used (for example: ATICE, 1% (7); DOLO-USI, 13% (113); Visual Analogic Scale, 14% (119)).

An analgo-sedation protocol was available for one-third of respondents (31%). A majority of respondents (86%) had a sedation scale available in their ICU, and 75% had one validated for use in the ICU including the Richmond Agitation Sedation Scale, the Sedation Agitation Scale, the Motor Activity Assessment Scale, and the Ramsay sedation scale. Frequency of use varied across respondents; 17% used them less than three times daily, while 53% used them less than six times daily. Amongst respondents not having a sedation scale, 85% were inclined to use one. The majority of respondents (93%) reported using sedation scales to assess the level of sedation. Reported use for dosage adjustment of sedatives was less frequent (73%). More than one-third of participants reported using sedation scales to assess pain and dosage adjustment of analgesics (37% and 33%, respectively).

One-third (31%) of respondents reported never using DSI, while 44% reported using it for <25% of their patients, 17% of respondents reported using it for 25 to 75% of patients and 5% reported using it for >75% of patients.

Pain was not evaluated in patients unable to self-report for 7% of respondents. Over 80% of respondents reported using physiological parameters or behaviors as proxies to identify pain in these patients. About one-half reported assessing pain during analgo-sedation breaks or after administration of analgesics. Only 11% of respondents used validated scales for these patients, such as the Behavioral Pain Scale and the Critical Care Pain Observation Tool. Interestingly, 13% of participants used DOLO-USI, an analgesia scale for patients unable to self-report, developed in Belgium [32].

### Barriers to optimal analgo-sedation practices

#### Common perceptions on the use of protocolized sedation and sedation scales

Common perceptions on sedation scales may challenge utilization (Table 4). Regarding the impact on autonomy, 14% of physicians agreed that using scales limits their autonomy. Only 68% of the nurses agreed that using these scales increase their autonomy and 66% that their use enhances their role. More physicians than nurses agreed that using sedation scales is associated with potential benefits, including increased autonomy for nurses and enhancement of their role, aid in monitoring administration of sedatives, and cost control. A majority (96%) of respondents reported a beneficial effect on patient outcomes. More physicians than nurses agreed that sedation scales actually influence sedatives’ prescriptions by physicians or administration by nurses. Finally, the perception that the level of sedation may be measured without using scales was present for over one-half (54%) of healthcare professionals. Knowledge and familiarity problems with using sedation scales and the perception that these are complex or that much time is required to use them were present for a minority of respondents.

**Table 4:** **Respondents’ agreement with statements reflecting common perceptions on sedation scales**

	**Total, % (** ***n*** **)** ^**a**^	**Physicians, % (** ***n*** **)** ^**b**^	**Nurses, % (** ***n*** **)** ^**c**^	***P*** **value** ^**d**^
**Effects**				
They make it possible to communicate better on the basis of objective numbers	86 (752)	91 (278)	84 (474)	0.001
They make it possible to make sedation practices consistent	84 (722)	92 (278)	80 (444)	<0.001
They restrict physicians’ autonomy	17 (145)	14 (42)	19 (103)	0.047
They give nurses more autonomy	73 (634)	82 (247)	68 (387)	<0.001
They enhance the nurses’ role	72 (601)	84 (249)	66 (352)	<0.001
They help to monitor the prescription of sedatives by physicians	48 (419)	52 (156)	47 (263)	0.098
They help to monitor the administration of sedatives by nurses	73 (634)	83 (252)	68 (382)	<0.001
They help to monitor costs	38 (318)	54 (160)	29 (158)	<0.001
They are useful for physicians	85 (740)	94 (285)	80 (455)	<0.001
They are not useful for nurses	9 (80)	9 (27)	9 (53)	0.504
**Uses**				
Using them influences the prescription of sedatives by physicians	77 (669)	89 (274)	70 (395)	<0.001
Using them influences the administration of sedatives by nurses	74 (640)	86 (266)	67 (374)	<0.001
Using them is beneficial for the patient	96 (848)	97 (300)	96 (548)	0.302
I can measure the level of sedation without using them	54 (455)	59 (173)	51 (282)	0.014
They are too complex for everyday use	10 (82)	16 (47)	6 (35)	<0.001
It doesn’t take much time if you use them every day	85 (724)	85 (251)	85 (473)	0.537
I don’t know any	7 (56)	9 (24)	6 (32)	0.106

Responses were provided in the form of a six-point Likert scale (‘Strongly disagree’, ‘Disagree’, ‘Inclined to disagree’, ‘Inclined to agree’, ‘Agree’, ‘Strongly agree’). The positive answers (‘Inclined to agree’, ‘Agree’, ‘Strongly agree’) were compiled in a one-and-only category and results are presented in the table. ^a^Number does not total 898 because not all respondents answered each item. ^b^Number does not total 323 because not all respondents answered each item. ^c^Number does not total 575 because not all respondents answered each item. ^d^Calculated using the chi-square test.

#### Common perceptions on the use of daily sedation interruption and reported contraindications

**Figure 2:**
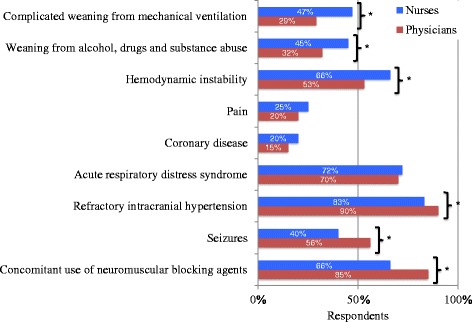
**Perceived contraindications to daily sedation interruption.** *Difference between groups is statistically significant (*P* < 0.05), *P* value calculated using the chi-square test.

Heterogeneity was present amongst physicians and nurses for reported contraindications to DSI (Figure 2). Contraindications primarily reported were refractory intracranial hypertension (85%, 703/824), concomitant management with neuromuscular blocking agents (73%, 600/824), acute respiratory distress syndrome (71%, 586/824) and hemodynamic instability (61%, 503/824).

Numerous common perceptions on DSI may challenge adherence (Table 5). These perceptions are systematically more present for nurses than physicians. Perceptions that DSI increases the risk of self-extubation, impairs patient comfort and creates traumatic memories were present. Clinicians’ projection of their own preferences not to have DSI if they were a patient was present for over one-half (57%) of healthcare professionals. Other potential barriers include organizational constraints, clinicians’ preferences for care (easier patient care), knowledge issues and poor outcome expectancy (in all patients or in lightly sedated patients). A majority of respondents reported that DSI should be performed on physicians’ orders only.

**Table 5:** **Respondents’ agreement with statements reflecting common perceptions of daily sedation interruption**

	**Total, % (** ***n*** **)** ^**a**^	**Physicians, % (** ***n*** **)** ^**b**^	**Nurses, % (** ***n*** **)** ^**c**^	***P*** **value** ^**d**^
DSI should only be performed on physicians’ orders	84 (716)	83 (254)	84 (472)	0.450
DSI increases the risk of complications such as self-extubation, pulling out of intravenous lines or feeding tubes …	78 (666)	69 (210)	82 (456)	<0.001
It is easier to take care of a sedated patient than a patient who is awake	71 (609)	54 (165)	80 (444)	<0.001
If I was intubated, I would prefer not to have my sedation stopped every day	57 (483)	41 (122)	65 (361)	<0.001
If patients are only lightly sedated, DSI is not useful	57 (482)	51 (155)	60 (327)	0.011
I don’t see the point of stopping sedation every day for every patient	54 (464)	48 (146)	57 (318)	0.006
DSI is detrimental to the comfort of intubated patients	52 (446)	37 (112)	60 (334)	<0.001
For organizational reasons, it is difficult to envisage DSI being performed for most of my patients	50 (427)	41 (126)	54 (301)	<0.001
DSI creates traumatic memories for the intubated patient	35 (294)	28 (84)	39 (210)	0.001
I’m not familiar with this practice	22 (175)	11 (33)	28 (142)	<0.001

Responses were provided in the form of a six-point Likert scale (’Strongly disagree’, ‘Disagree’, ‘Inclined to disagree’, ‘Inclined to agree’, ‘Agree’, ‘Strongly agree’). The positive answers (‘Inclined to agree’, ‘Agree’, ‘Strongly agree’) were compiled in a one-and-only category and results are presented in the table. DSI, daily sedation interruption. ^a^Number does not total 898 because not all respondents answered each item. ^b^Number does not total 323 because not all respondents answered each item. ^c^Number does not total 575 because not all respondents answered each item. ^d^Calculated using the chi-square test.

## Discussion

Our data indicate poor compliance with analgo-sedation recommendations in terms of assessment of pain and sedation, protocolized sedation and DSI. We identified barriers potentially impairing adherence, with differences observed between physicians and nurses.

Despite recognized benefits, significant gaps in assessment of sedation and pain are present. Regarding pain assessment in patients unable to self-report, behavioral scales (Behavioral Pain Scale, Critical Care Pain Observation Tool) are scarcely available, leaving a vast majority (88%) of respondents relying only on behaviors and/or physiological parameters, even though the latter are unreliable pain surrogates [33,34]. Sedation scales are widely adopted, as availability is similar to the highest frequencies reported in the international context [35,36]. Unfortunately, the most valid and reliable scales (Sedation Agitation Scale, Richmond Agitation Sedation Scale) are available to only 27% of respondents [1]. Additionally, whether a daily frequency of use below three times a day, as indicated by 17% of respondents, is sufficient to impact patient outcomes is questionable [1]. Protocolized analgo-sedation, combining patient assessment and algorithms by which nurses adjust analgesics’ and sedatives’ dosages accordingly, has been associated with beneficial patient outcomes including reduced duration of mechanical ventilation [3]. We found that protocols are available to only one-third of respondents, comparing unfavorably with the highest rates of availability reported in the United Kingdom (80%) and the United States (71%) [35,36]. Convenience sampling of the latter surveys may explain these results, as only clinicians highly involved in analgo-sedation practices may have responded. Because assessment is essential in providing the basis for the treatment of pain and agitation/anxiety, underuse of appropriate scales may lead to inappropriate treatment of pain and agitation. Also, the high availability of sedation scales in Belgian ICUs contrasts with the low adoption of protocolized sedation, potentially increasing treatment delays.

Perceptions challenging utilization of sedation scales are present, especially for nurses. For example, more nurses than physicians disagree that using a sedation scale may: increase nurses’ autonomy; enhance their role; or influence management beyond simple assessment, with an effect on physicians’ prescription or nurses’ administration of sedatives. These findings show that nurse empowerment has not been appropriately tackled in units where sedation scales are used. We hypothesize that a low availability of protocols clarifying nurses’ responsibilities as well as how to adapt dosages according to assessments may explain poor utilization of scales. More physicians than nurses agreed with statements reporting potential benefits of sedation scales (improve communication, consistency in sedation practices and help to monitor cost). However, although statistically significant, some of these differences between physicians and nurses are small and questionable of clinical relevance.

DSI is infrequently used in Belgium, despite substantial clinical benefits including reduced length of stay [2,4]. Internationally, less than one-half of clinicians report using DSI [11,37-39]. Our results contrast with those of a recent survey showing that one-half of the respondents reported regular use of DSI (defined as greater than 75% of mechanically ventilated patients) [21]. Highly motivated respondents may explain the latter results, as these were part of a statewide ICU quality improvement collaborative.

Perceptions challenging DSI are present for more nurses than physicians. Previous research showed lack of nursing acceptance is one of the three most common barriers to the practice [14]. Some of our findings may explain such reluctance. First, safety concerns are present for more nurses than physicians (perception that DSI increases the risk of complications such as device removal, impairs patient comfort and creates traumatic memories). These fears were reported previously, despite evidence demonstrating safety of DSI [14,20,21,6,40-42]. Accordingly, more nurses than physicians agree they would prefer not to have DSI if they were a patient. Second, more physicians than nurses find DSI is contraindicated in situations where sedation is used for reasons other than agitation (concomitant management with neuromuscular blocking agents, seizures and refractory intracranial hypertension). In contrast, more nurses than physicians find that situations frequently encountered in the ICU (hemodynamic instability, complicated weaning from mechanical ventilation and substance abuse) are contraindications to DSI, and therefore many patients may not benefit from this strategy. The perception that complicated weaning from mechanical ventilation is a contraindication to DSI is particularly worrisome because the process of discontinuing ventilator support is affected by sedative use. DSI facilitates extubation by increasing the likelihood that patients are neurologically ready for it, once respiratory failure has improved, and combination with spontaneous breathing trials, has improved 1-year survival in ICU patients [2]. Appropriateness of performing DSI in specific patients remains controversial, because the benefits of alcohol withdrawal or ongoing agitation remain uncertain and efficacy and/or safety have not yet been sufficiently demonstrated in surgical, trauma and neurologic patients [1,5,43]. Yet some contraindications to the practice are not subject to debate, such as concomitant use of neuromuscular blocking agents. However, only 66% of nurses and 85% of physicians report the latter as a contraindication to DSI, which reflects a concerning lack of knowledge. Interestingly, strategies successful in implementing DSI have used safety screens and failure criteria, ensuring empowerment of ICU staff, particularly nurses, to assess whether DSI may be safely performed in their patients [2,23]. Also, favorable interdisciplinary communication with sedation goals discussed on rounds increases nurse’s willingness to perform DSI [17,21]. Finally, perceptions including that an asleep patient is easier to take care of than an awake patient and that DSI use is limited by organizational constraints are also present for more nurses than physicians. Perceived impact on workload by nurses has been shown to be higher for DSI than protocolized sedation [5]. The perception that DSI is hard work was shown to be negatively associated with regular use [21]. Incentives may include the perspective that awake, alert, comfortable patients may actually be easier to take care of for various reasons. First, the risk of delirium may be reduced. Second, patients may communicate their needs to ICU staff and participate in their own care decisions, even though communication remains challenging. Education highlighting these incentives for nurses, and availability of appropriate tools to enhance communication with nonverbal patients (for example, communication boards, illustrations) may improve nurses’ acceptance of the practice.

### Scope of the study (limitations and strengths)

Our survey has limitations inherent to this methodology. First, responder bias is an issue, because relying on self-report probably overestimates use of evidence-based practices as compared with actual practices. A recent study compared survey and observational data for analgo-sedation practices in the ICU and showed that DSI was reported to be 66% but was observed in only 36% of patients [44]. In our study, the reported frequency of use for the strategy was significantly higher for physicians than for nurses (*P* < 0.001). The actual frequency of use may therefore be lower than reported by physicians, as nurses are those working at the bedside. However, our study focused on perceptions of different clinicians, rather than actual practices. Additionally, contrasting with other surveys, our very large sampling frame included clinicians in various positions (not only managerial positions); therefore our results probably reflect bedside practices [11]. Second, nonresponder bias may occur; however, our response rate was high, as was the number of respondents (*n* = 898). Additionally, comparison between hospital characteristics of responders and nonresponders did not show significant differences.

Diversity in participants’ background and position, as well as high respondents’ and institutional response rates, increase the generalizability of our results. However, our study was conducted in Belgium and results may not be fully applicable to other countries. Other strengths of our survey deserve to be mentioned. First, we achieved response rates above the expected 50%, allowing adequate power to analyze each profession, and conclusions on barriers and enablers may be drawn for each population individually. Second, in contrast to most studies, we did not rely on convenience sampling, as we avoided using existing contact databases of professional societies most likely to generate selection bias. Our sampling frame was carefully created as a census of physicians (all Belgian physicians were surveyed) and a probabilistic sampling of nurses, across all Belgian hospitals, through their HRDs. This further reduced responders’ selection bias. Third, the survey instrument was created involving a multidisciplinary team for its construction and to ensure face and content validity. Additionally, various pretesting methods were combined to improve the instrument (respondent debriefings, test–retest reliability).

## Conclusion

Compliance with analgesia and sedation recommendations in Belgian ICUs is poor for assessment of analgesia and sedation and strategies used to target light sedation (DSI and protocolized sedation). Numerous misperceptions hinder compliance with these strategies. Frequency of agreement with such misperceptions is systematically higher for nurses than for physicians. Implementational strategies must include efficient nurse empowerment and protocolized care to garner buy-in from key stakeholders in pain and sedation scales, while education on safety and contraindications of DSI must be discussed and agreed upon in ICU teams.

## Key messages

- Despite beneficial patient outcomes, the use of strategies targeting light sedation in the ICU leaves room for improvement. Sedation scales are available to 86% of clinicians, but frequency of use remains low (17% used them less than three times daily, while 53% used them less than six times daily). DSI is infrequently used; 75% of clinicians use it for <25% of their patients.
- More physicians than nurses agree upon potential benefits of using sedation scales, including increased autonomy for nurses and enhancement of their role, aid in monitoring administration of sedatives and cost control.
- Nurses report situations frequently encountered in the ICU (hemodynamic instability, complicated weaning from mechanical ventilation and substance abuse) as contraindications to DSI more often than physicians. Local protocols must consider clearly stating patients who should (not) receive DSI.
- Common misperceptions of DSI, potentially impairing adherence, are systematically more present for nurses than for physicians, including the perception that DSI increases the risk of self-extubation, impairs patient comfort and creates traumatic memories.
- For patients unable to self-report, over 80% of clinicians report use of physiological parameters or behaviors as proxies to identify pain. Only 12% use validated scales for these patients, including the Behavioral Pain Scale and the Critical Care Pain Observation Tool.

## Additional file

Additional file 1:
**Shows the survey tool (French version).**

## Abbreviations

DSI: daily sedation interruption
HRD: human resources’ department
