# Supplementary material for: Current practices and barriers impairing physicians’ and nurses’ adherence to analgo-sedation recommendations in the intensive care unit - a national survey
Source: Crit Care. 2014 Dec 5;18(6):655. doi: 10.1186/s13054-014-0655-1 (PMC4324789; doi:10.1186/s13054-014-0655-1)
Supplement: Additional file 1: — Shows the survey tool (French version). [file 13054_2014_655_MOESM1_ESM.doc]

**Additional file 1 : Survey tool (paper version, french).**

**A. Disponibilité des protocoles et échelles de sédation.**

Par **"protocoles de sédation"**, on entend des instructions et/ou un algorithme de traitement permettant aux médecins et/ou aux infirmiers d'ajuster les doses de sédatifs aux besoins du patient.

Par **"échelles de sédation"**, on entend un outil permettant d'évaluer le niveau de sédation. Par exemple: l'échelle de Ramsay, l'échelle SAS, l'échelle de Richmond (Richmond Agitation Sedation Scale, RASS)...

------------------------------------------------------------------------------------------------------------------------

1. Un **protocole écrit** de sédation est il **disponible** pour les soignants, dans votre service?

❑ Oui ❑ Non ❑ Je ne sais pas

Si vous avez répondu **"non"** ou **"je ne sais pas"** à la question n°1, veuillez ne pas répondre à la question n°2 et **passer à la question n°3**.

1. Veuillez indiquer la **fréquence moyenne** à laquelle vous utilisez ce protocole?

❑ Jamais

❑ Occasionnellement (<1x/jour)

❑ Quotidiennement (1x/jour)

❑ Fréquemment (>1x/jour)

1. Une **échelle de sédation** est elle **disponible** pour les soignants, dans votre service?

❑ Oui ❑ Non ❑ Je ne sais pas

Si vous avez répondu **"oui"** à la question n°3, **passez directement à la page 3.**

Si vous avez répondu **"non"** ou **"je ne sais pas"** à la question n°3, **passez à la page 2.**

**B. Utilisation des échelles de sédation.**

Par **"échelles de sédation"**, on entend un outil permettant d'évaluer le niveau de sédation. Par exemple: l'échelle de Ramsay, l'échelle SAS, l'échelle de Richmond (Richmond Agitation Sedation Scale, RASS)...

--------------------------------------------------------------------------------

Répondre aux questions de cette page que si votre service **ne dispose PAS** d’échelles de sédation.

1. Etes-vous **favorable** à l'utilisation d'une échelle de sédation au sein de votre service?

❑ Oui ❑ Non ❑ Je ne sais pas

1. **A quoi vous servirait** une échelle de sédation?

Cochez **tout ce qui est applicable**.

❑ A évaluer la sédation du patient

❑ A évaluer la douleur du patient

❑ A adapter les doses de sédatifs

❑ A adapter les doses d'analgésiques

❑ Je ne sais pas à quoi servirait cette échelle

❑ L'échelle ne servirait à rien

❑ Autre (veuillez préciser svp) : _______________________________

**Passez directement à la page 4.**

**B. Utilisation des échelles de sédation.**

Par **"échelles de sédation"**, on entend un outil permettant d'évaluer le niveau de sédation. Par exemple: l'échelle de Ramsay, l'échelle SAS, l'échelle de Richmond (Richmond Agitation Sedation Scale, RASS)...

--------------------------------------------------------------------------------

Répondre aux questions de cette page que si votre service **dispose** d’une échelle de sédation.

1. Au sein de votre service, **quelle(s) échelle(s)** est (sont) utilisée(s) pour mesurer le niveau de sédation?

Cochez **tout ce qui est applicable**.

❑ Motor Activity Assessment Scale (MAAS)

❑ Ramsay sedation scale (échelle de Ramsay)

❑ Richmond Agitation Sedation Scale (RASS)

❑ Sedation Agitation Scale (SAS ou échelle de Riker)

❑ GCS (Glasgow Coma Scale)

❑ Autre (veuillez préciser svp) : _______________________________

1. **A quoi vous sert** l'échelle de sédation du service?

Cochez **tout ce qui est applicable**.

❑ A évaluer la sédation du patient

❑ A évaluer la douleur du patient

❑ A adapter les doses de sédatifs

❑ A adapter les doses d'analgésiques

❑ Je ne sais pas à quoi sert cette échelle

❑ L'échelle ne sert à rien

❑ Autre (veuillez préciser svp) : _______________________________

1. Pour chaque patient sédaté, à quelle **fréquence minimale** l'échelle de sédation du service est elle utilisée?

❑ Jamais

❑ < 1x / jour

❑ 1x / jour

❑ 3x / jour (soit 1x/8h)

❑ 6x / jour (soit 1x/4h)

❑ 1x / heure

❑ Autre (veuillez préciser svp) : _______________________________

**B. Utilisation des échelles de sédation.**

1. A propos de l**'utilisation** des échelles de sédation...

... Êtes-vous **d'accord** ou **pas d'accord** avec les affirmations suivantes?

|  | Pas du tout d'accord | Pas d'accord | Plutôt pas d'accord | Plutôt d'accord | D'accord | Tout à fait d'accord |
| --- | --- | --- | --- | --- | --- | --- |
| *Les utiliser est bénéfique pour le patient* | O | O | O | O | O | O |
| *Les utiliser influence la prescription de sédatifs par les médecins* | O | O | O | O | O | O |
| *Je n'en connais aucune* | O | O | O | O | O | O |
| *Je peux mesurer le niveau de sédation sans les utiliser* | O | O | O | O | O | O |
| *Les utiliser quotidiennement est rapide* | O | O | O | O | O | O |
| *Elles sont trop complexes pour une utilisation quotidienne* | O | O | O | O | O | O |
| *Les utiliser influence l'administration de sédatifs par les infirmiers* | O | O | O | O | O | O |
| *Je sais comment les utiliser* | O | O | O | O | O | O |

1. A propos des **effets** des échelles de sédation...

... Êtes-vous **d'accord** ou **pas d'accord** avec les affirmations suivantes?

|  | Pas du tout d'accord | Pas d'accord | Plutôt pas d'accord | Plutôt d'accord | D'accord | Tout à fait d'accord |
| --- | --- | --- | --- | --- | --- | --- |
| *Elles permettent de mieux communiquer sur base de chiffres objectifs* | O | O | O | O | O | O |
| *Elles permettent d'homogénéiser les pratiques de sédation* | O | O | O | O | O | O |
| *Elles limitent l'autonomie des médecins* | O | O | O | O | O | O |
| *Elles servent à contrôler les coûts* | O | O | O | O | O | O |
| *Elles servent à contrôler l'administration de sédatifs par les infirmiers* | O | O | O | O | O | O |
| *Elles ne sont pas utiles pour les infirmiers* | O | O | O | O | O | O |
| *Elles valorisent le rôle des infirmiers* | O | O | O | O | O | O |
| *Elles sont utiles pour les médecins* | O | O | O | O | O | O |
| *Elles servent à contrôler la prescription de sédatifs par les médecins* | O | O | O | O | O | O |
| *Elles donnent plus d'autonomie aux infirmiers* | O | O | O | O | O | O |

**C. Arrêts journaliers de sédation.**

Par "**arrêt journalier de sédation**" (="AJS" ci après), on entend l'interruption des **perfusions continues** de sédatifs et d'analgésiques, **au moins une fois par jour**, jusqu'au réveil du patient. La perfusion peut être redémarrée, si nécessaire, après évaluation de son utilité.

1. A propos des "AJS"(= arrêts journaliers de sédation)...

...Etes-vous **d'accord** ou **pas d'accord** avec les affirmations suivantes?

|  | Pas du tout d'accord | Pas d'accord | Plutôt pas d'accord | Plutôt d'accord | D'accord | Tout à fait d'accord |
| --- | --- | --- | --- | --- | --- | --- |
| *Les AJS nuisent au confort du patient intubé* | O | O | O | O | O | O |
| *Pour des raisons d'organisation, il est compliqué d'envisager un AJS, chez la majorité de mes patients* | O | O | O | O | O | O |
| *Les AJS créent des souvenirs traumatisants chez le patient intubé* | O | O | O | O | O | O |
| *Je ne vois pas l'intérêt d'arrêter la sédation tous les jours, chez tous les patients* | O | O | O | O | O | O |
| *Si j'étais intubé, je préfèrerais que l’on n’arrête pas ma sédation tous les jours* | O | O | O | O | O | O |
| *Les AJS doivent être effectués uniquement sur ordre médical* | O | O | O | O | O | O |
| *Il est plus facile de s'occuper d'un patient sédaté que d'un patient éveillé* | O | O | O | O | O | O |
| *Je ne connais pas cette pratique* | O | O | O | O | O | O |
| *Les AJS augmentent le risque de complications telles que les auto-extubations, les arrachages de sondes ou de voies intraveineuses...* | O | O | O | O | O | O |
| *Si les patients sont peu sédatés, il n'est pas utile de faire un AJS* | O | O | O | O | O | O |

1. En prenant comme période de référence **le dernier jour ou vous avez travaillé**, pourriez vous indiquer le **nombre total** de patients qui étaient sous votre responsabilité ce jour là?

Si vous êtes **médecin chef** ou **infirmier chef**, veuillez répondre pour **tous** les patients du service.

- le nombre total de patients sous votre responsabilité : _____

- - **Parmi ces patients**, veuillez indiquer:

- le nombre de patients sous perfusions continues de sédatifs et/ou d'opiacés : _____

- le nombre de patients où un arrêt journalier de sédation a été effectué, avec reprise par la suite : _____

- le nombre de patients où la sédation a été arrêtée, sans reprise par la suite : _____

1. D'après **votre expérience personnelle**, chez quel type de patients faut-il **éviter** un "arrêt journalier de sédation"?

Cochez **tout ce qui est applicable**.

❑ Il n'y a pas de contre-indication à cette pratique

❑ Patient non extubable

❑ Patient en ARDS (Acute Respiratory Distress Syndrome)

❑ Patient coronarien

❑ Patient en sevrage (alcool, substances illicites, médicaments)

❑ Patient avec hypertension intracrânienne non contrôlée

❑ Patient avec épilepsie, convulsions

❑ Patient hémodynamiquement instable

❑ Patient douloureux

❑ Patients curarisés

❑ Autre (veuillez préciser ci-dessous svp)

**D. Pratiques de sédation.**

Par **"arrêt journalier de sédation"** (="AJS" ci après), on entend l'interruption des perfusions continues de sédatifs et d'analgésiques, au moins une fois par jour, jusqu'au réveil du patient. La perfusion peut être redémarrée, si nécessaire, après évaluation de son utilité.

Par **"sédatifs"** on entend le propofol (=DIPRIVAN) et les benzodiazépines telles que le lorazepam (=TEMESTA), le midazolam (=DORMICUM).

Par **"opiacés"** on entend les molécules telles que la morphine, le fentanyl, le sufentanil (=SUFENTA), le remifentanil (=ULTIVA), l'alfentanil (=RAPIFEN), le piritramide (=DIPIDOLOR).

Par **"paralysants neuromusculaires"**, on entend les agents curarisants tels que le rocuronium (=ESMERON), le cisatracurium (=NIMBEX), le vecuronium (=NORCURON), l'atracurium (=TRACRIUM), le mivacurium (=MIVACRON)

1. Dans votre unité de soins, quel pourcentage des patients reçoivent des

**perfusions continues de sédatifs**, à un moment de leur séjour ?

❑ > 75% ❑ 25-75% ❑ < 25% ❑ Aucun patient

1. **Parmi ces patients** (recevant des sédatifs en perfusion continue), pour quel

pourcentage un "**arrêt journalier de sédation**" est il réalisé?

❑ Aucun patient ❑ < 25% ❑ 25-75% ❑ > 75%

1. **Lorsqu'un "arrêt journalier de sédation" est réalisé**, les perfusions continues d'**opiacés** sont elles aussi interrompues?

❑ Oui ❑ Non

1. Pour chacun des motifs suivants, veuillez indiquer la **fréquence d'utilisation des sédatifs**:

|  | Très fréquemment | Fréquemment | Rarement | Jamais |
| --- | --- | --- | --- | --- |
| *Favoriser l'amnésie (éviter les "mauvais" souvenirs du séjour)* | O | O | O | O |
| *Faciliter le transport des patients* | O | O | O | O |
| *Traiter le délirium des soins intensifs* | O | O | O | O |
| *Faciliter la toilette du patient* | O | O | O | O |
| *Faciliter les actes invasifs courts (pose de cathéters, drains...)* | O | O | O | O |
| *Faciliter les aspirations endotrachéales* | O | O | O | O |
| *Traiter la douleur* | O | O | O | O |
| *Faciliter les soins de plaies* | O | O | O | O |
| *Traiter l'insomnie* | O | O | O | O |
| *Faciliter l'intubation* | O | O | O | O |
| *Faciliter la ventilation mécanique* | O | O | O | O |

1. **Dans votre service**, pour quel pourcentage de patients utilise-t'on les régimes d'analgo-sédation suivants?

|  | >75 % des patients | 25-75 % des patients | <25 % des patients | Jamais |
| --- | --- | --- | --- | --- |
| Midazolam (=DORMICUM) sans opiacés | O | O | O | O |
| Midazolam (=DORMICUM) + opiacés | O | O | O | O |
| Propofol (=DIPRIVAN) sans opiacés | O | O | O | O |
| Propofol (=DIPRIVAN) + opiacés | O | O | O | O |
| Opiacés seuls | O | O | O | O |
| Autre régime (s) de sédation (préciser svp) : | O | O | O | O |

1. **Dans votre service**, pour quel pourcentage de patients ajoute-t-on des

co-analgésiants aux médicaments proposés ci-dessus?

|  | >75 % des patients | 25-75 % des patients | <25 % des patients | Jamais |
| --- | --- | --- | --- | --- |
| + Clonidine (=CATAPRESSAN) | O | O | O | O |
| + Kétamine (=KETALAR) | O | O | O | O |

1. Lorsque la voie intraveineuse est utilisée, **de quelle manière** administrez vous les médicaments suivants?

|  | Principalement « **perfusion continue »**  (+- bolus) | Principalement « **bolus seuls »** | Médicaments non utilisés |
| --- | --- | --- | --- |
| Midazolam (=DORMICUM) | O | O | O |
| Morphine | O | O | O |
| Fentanyl | O | O | O |
| Sufentanil (=SUFENTA) | O | O | O |
| Alfentanil (=RAPIFEN) | O | O | O |
| Remifentanil (=ULTIVA) | O | O | O |
| Piritramide (=DIPIDOLOR) | O | O | O |
| Clonidine (=CATAPRESSAN) | O | O | O |

1. Quel pourcentage de vos patients reçoivent des **paralysants neuromusculaires** durant leur séjour (en dehors de ceux administrés lors de l’intubation) ?

❑ > 75%

❑ 51-75%

❑ 26-50%

❑ 10-25%

❑ <10%

❑ Jamais

**E. Pratiques d’analgésie.**

Par **"opiacés"** on entend les molécules telles que la morphine, le fentanyl, le sufentanil (=SUFENTA), le remifentanil (=ULTIVA), l'alfentanil (=RAPIFEN), le piritramide (=DIPIDOLOR).

1. **Dans votre service**, pour quel pourcentage de patients utilise-t-on les régimes d’analgésie suivants ?

|  | >75 %  des patients | 25-75 %  des patients | <25 %  des patients | Jamais |
| --- | --- | --- | --- | --- |
| Opiacés en intra-veineux | O | O | O | O |
| Péridurales | O | O | O | O |
| PCA  (= "Patient controlled analgesia") | O | O | O | O |

1. **Chez les patients sédatés**, comment évaluez-vous la **douleur**?

Cochez **tout ce qui est applicable**.

❑ Nous n'évaluons pas la douleur chez le patient sédaté

❑ Nous monitorons les paramètres physiologiques (tension artérielle, poul, fréquence respiratoire...)

❑ Nous observons les comportements (mouvements, expression faciale, posture...)

❑ Nous évaluons la douleur lors des arrêts d'analgo-sédation

❑ Nous observons les modifications des paramètres physiologiques suite à l'administration d'analgésiques

❑ Nous utilisons l'échelle BPS (=Behavioural Physiological Pain Scale)

❑ Nous utilisons l'échelle CPOT (=Critical Care Pain Observational Tool)

❑ Nous utilisons l'échelle DOLO-USI

❑ Autre (veuillez préciser ci dessous)

**F. Données démographiques personnelles et du service.**

Par USI, on entend unité de soins intensifs.

- Quelle est votre fonction **actuelle**?

❑ Médecin **chef de service** de l'USI

❑ Médecin du **staff permanent** à l'USI

❑ Médecin à **temps partiel** à l'USI

❑ **Assistant** (en cours de formation)

❑ Autre type de médecin (préciser svp)

❑ Infirmier **chef de service** de l'USI

❑ Infirmier du **staff permanent** à l'USI

❑ Infirmier à **temps partiel** à l'USI

❑ Autre type d'infirmier (préciser svp)

Précisions - Autre type de médecin ou infirmier : _____________________________

**Si vous êtes médecin, passez directement à la page 13.**

**Si vous êtes infirmier, passez à la page suivante.**

**Données personnelles et du service (Infirmiers).**

Les questions de cette page sont à compléter par les **infirmiers uniquement**.

1. Veuillez préciser votre type de **formation**.

Cochez le **dernier diplôme en date**.

❑ Infirmier breveté

❑ Infirmier gradué ou bachelier

❑ Infirmier SISU

❑ Autre (veuillez préciser svp) : _______________________________

1. Veuillez préciser votre **nombre d'années d'expérience aux soins intensifs**.

❑ < 2 ans

❑ 2-5 ans

❑ 6-10 ans

❑ 11-20 ans

❑ > 20 ans

1. Dans quel **type de service** de soins intensifs exercez vous?

❑ Médical

❑ Chirurgical

❑ Médico-chirurgicale

1. Veuillez indiquer les catégories diagnostiques **les plus fréquentes** des patients admis dans votre service?

Cochez **tout ce qui est applicable**.

❑ Neurologie

❑ Pneumologie

❑ Cardiologie

❑ Gastro-entérologie

❑ Hématologie

❑ Infectieux

❑ Traumatologie

❑ Grands brûlés

❑ Autre (veuillez préciser)

1. Veuillez cocher **l'horaire de travail** qui vous correspond le mieux.

❑ Je travaille essentiellement de jour

❑ Je travaille essentiellement de nuit

❑ Je travaille autant de jour que de nuit

❑ Autre (veuillez préciser svp) : _______________________________

**L’enquête est terminée. Passez directement à la page 14.**

**Données personnelles et du service (Médecins).**

Les questions de cette page sont à compléter par les **médecins uniquement**.

1. Veuillez préciser votre **formation** de base?

Cochez le **dernier diplôme en date**.

❑ Anesthésiste

❑ Interniste

❑ Cardiologue

❑ Assistant anesthésiste (formation en cours)

❑ Assistant interniste (formation en cours)

❑ Assistant cardiologue (formation en cours)

❑ Autre (veuillez préciser svp) : __________________________

1. Veuillez préciser votre **nombre d'années d'expérience aux soins intensifs**.

❑ < 2 ans

❑ 2-5 ans

❑ 6-10 ans

❑ 11-20 ans

❑ > 20 ans

1. Dans quel **type de service** de soins intensifs exercez vous?

Cochez **tout ce qui est applicable**.

❑ Médical

❑ Chirurgical

❑ Médico-chirurgicale

1. Veuillez indiquer les **catégories diagnostiques** les plus fréquentes des patients admis dans votre service?

❑ Neurologie

❑ Pneumologie

❑ Cardiologie

❑ Gastro-entérologie

❑ Hématologie

❑ Infectieux

❑ Traumatologie

❑ Grands brûlés

❑ Autre (veuillez préciser svp) : _______________________________

1. En moyenne, quelle proportion de vos patients...

...sont admis après une **chirurgie élective**? _____

(La **chirurgie élective** recouvre les interventions qui peuvent normalement être programmées, le délai ne mettant pas en danger la santé du patient.)

...nécessitent de la **ventilation mécanique**? ____

L'enquête est maintenant terminée! **Nous vous remercions de votre participation.**

Il vous suffit maintenant de nous **renvoyer le questionnaire** **complété** dans **l’enveloppe timbrée jointe** qui vous a été fournie à cet effet.

Vos remarques/commentaires concernant l'enquête sont les bienvenu(e)s.

Merci de les noter ci-dessous :

Désirez-vous recevoir les résultats de l'enquête?

❑ Oui ❑ Non

Si vous désirez recevoir les résultats, veuillez indiquer votre adresse courriel ci-dessous :

___________________________________________________________________
